# Tracking Contributions to Human Body Burden of Environmental Chemicals by Correlating Environmental Measurements with Biomarkers

**DOI:** 10.1371/journal.pone.0093678

**Published:** 2014-03-28

**Authors:** Hyeong-Moo Shin, Thomas E. McKone, Michael D. Sohn, Deborah H. Bennett

**Affiliations:** 1 Department of Public Health Sciences, University of California Davis, Davis, California, United States of America; 2 Environmental Energy Technologies Division, Lawrence Berkeley National Laboratory, Berkeley, California, United States of America; 3 School of Public Health, University of California, Berkeley, California, United States of America; Florida International University, United States of America

## Abstract

The work addresses current knowledge gaps regarding causes for correlations between environmental and biomarker measurements and explores the underappreciated role of variability in disaggregating exposure attributes that contribute to biomarker levels. Our simulation-based study considers variability in environmental and food measurements, the relative contribution of various exposure sources (indoors and food), and the biological half-life of a compound, on the resulting correlations between biomarker and environmental measurements. For two hypothetical compounds whose half-lives are on the order of days for one and years for the other, we generate synthetic daily environmental concentrations and food exposures with different day-to-day and population variability as well as different amounts of home- and food-based exposure. Assuming that the total intake results only from home-based exposure and food ingestion, we estimate time-dependent biomarker concentrations using a one-compartment pharmacokinetic model. Box plots of modeled R^2^ values indicate that although the R^2^ correlation between wipe and biological (e.g., serum) measurements is within the same range for the two compounds, the relative contribution of the home exposure to the total exposure could differ by up to 20%, thus providing the relative indication of their contribution to body burden. The novel method introduced in this paper provides insights for evaluating scenarios or experiments where sample, exposure, and compound variability must be weighed in order to interpret associations between exposure data.

## Introduction

Correlation coefficients between environmental and biomarker measurements are widely used in environmental health assessments and epidemiology to explain the exposure associations between environmental media and human body burdens [1]–[4]. As a result considerable attention and effort have been given to interpretation of these coefficients [5]–[7]. However, there is limited information available on how the variance in environmental measurements, the relative contribution of exposure sources, and the elimination half-life affect the reliability of the resulting correlation coefficients. To address this information gap, we conducted a simulation study for various exposure scenarios of home-based exposure (e.g., inhalation, dermal uptake, non-dietary dust ingestion) to explore the impacts of pathway-specific scales of exposure variability on the resulting correlation coefficients between environmental and biomarker measurements.

Biomonitoring data, including those from blood, urine, hair, etc., have been used extensively to identify and quantify human exposures to environmental and occupational contaminants [8], [9]. However, because the measured levels in biologic samples result from multiple sources, exposure routes, and environmental media, the levels mostly fail to reveal how the exposures are linked to the source or route of exposure [10]. Thus, comparison of biologic samples with measurements from a single environmental medium (e.g., dust or air) results in weak correlations and lacks statistically significance. In addition, cross-sectional biological sample sets that track a single marker have large population variability and do not capture longitudinal (i.e. day-to-day) variability, especially for compounds with relatively short biologic half-lives, which can be on the order of days such as pesticides and phthalates. Therefore, in the case where the day-to-day variability of biological sample measurements is large, the use of biomarker samples with a low number of biological measurements in epidemiologic studies as a dependent variable can result in a misclassification of exposure as well as questions of reliability [11].

For chemicals frequently found at higher levels in indoor residential environments than in outdoor environments, it is common to assume that major contributions to cumulative intake are home-based exposure and/or food ingestion. This simplification can be further justified because people generally spend more than 70 percent of their time indoors [12], [13]. Compounds with significant indoor sources and long half-lives in the human body–on the order of years for chemicals such as polybrominated diphenyl ethers (PBDEs)–have been found to have positive associations between indoor dust or air concentrations and serum concentrations in U.S. populations [4], [14]–[17]. On the other hand, extant research has not reported significant associations between indoor samples and biomarkers for chemicals primarily associated with food-based exposures, for example, bisphenol-A [18] and perfluorinated compounds [19]. For chemicals with both home- and food-based exposure pathways and short body half-lives (on the order of days), as is the case for many pesticides, a significant association between indoor samples and biomarkers is found less frequently or relatively weak compared to PBDEs [1], [20]–[23]. To better interpret these types of findings, we provide here a simulation study for various exposure scenarios to explore the role of the chemical properties and exposure conditions that are likely to give rise to a significant contribution from indoor exposures. We then assess for these situations the magnitude and variance of the associated correlation coefficients between biomarker and indoor levels.

The objectives of this study are (1) to generate simulated correlation coefficients between environmental measurements and biomarkers with different contributions of home-based exposure to total exposure and different day-to-day and population variability of intake from both residential (home) environments and food, (2) to interpret the contribution of home-based exposure to human body burden for two hypothetical compounds whose half-lives are on the order of days and years, and (3) to determine how the pattern of variability in exposure attributes impacts the resulting correlation coefficients linking biomarker levels to exposure media concentrations.

## Materials and Methods

### 2. 1. Overview

In this study, our first step is to synthetically generate daily environmental concentrations and food exposure concentrations based on variations of day-to-day intake from residential environments and food as well as different relative contributions of home-based and food-based exposure. As different chemicals are likely to have different relative contributions from the home-based and food-based exposure pathways, we conducted our simulations across the full range of relative contributions between the two pathways to address all plausible scenarios for various compounds. We combine the simulated home-based exposures associated with indoor environmental concentrations and food concentrations, assuming that the total intake results only from home-based exposure and food ingestion. From these inputs we estimate time-dependent biomarker concentrations using a one-compartment pharmacokinetic model. We then computed correlation coefficients between simulated environmental and biomarker concentrations.

In order to facilitate numerous simulations, several simplifications are made regarding (1) a representative environmental medium for home exposure, (2) a distribution of environmental (inhalation/dermal) and food intake, and (3) sources of exposure. First, we select chemical concentrations from indoor wipe samples (C_wipe_) as a way to represent home-based exposures that result from all potential exposure routes, including inhalation, non-dietary dust ingestion, and dermal uptake. From these wipe concentrations, resulting home-based exposure (E_home_) can be assumed to be linearly related to C_wipe_ and E_home_ and C_wipe_ are assumed for simplicity to be equal. In addition, we assume that a contaminated food intake rate represents food exposures (E_food_). Second, we select C_wipe_ and E_food_ from log-normal distributions of variability across both population and time [6]. Lastly, we assume that the total intake accounting for biomonitoring data results from E_home_ and E_food_, excluding any other exposure pathways.

Calculating the correlation coefficient between environmental and biomarker measurements requires a number of steps. First, we generate synthetic wipe concentrations for a subject's home *i* on a day 1 (C_wipe,i,1_) and food exposure for a subject *i* on a day 1 (E_food,i,1_). Second, we generate a wipe concentration for a subject's home *i* on a given day *j* (C_wipe,i,j_) by correlating it with a wipe sample on the previous day (C_wipe,i,j-1_). We then apply this approach for generating C_wipe,i,j_ to generate synthetic food exposures for a subject *i* on a given day *j* (E_food,i,j_). Third, we vary the contribution of home exposure to total exposure (X_1_) to generate a different contribution of home and food exposures, based on the assumption that E_home_ is linearly related and equal to C_wipe_. Fourth, we add E_home,i,j_ and E_food,i,j_ for a total daily intake rate for a subject *i* on a given day *j*. Fifth, time-dependent biological concentrations are estimated using a one-compartment pharmacokinetic model. Finally, we compute Pearson's correlation coefficients between wipe and biological (e.g., serum) concentrations for our simulated population of 500 on each of 30 days.

### 2. 2. Monte Carlo Simulations

#### 2.2.1. Simulated home and food exposures

We assumed that wipe concentrations across the population are log-normally distributed with mean (μ_wipe_ = 1.0 µg/g) and standard deviation expressed as a coefficient of variation (CV_wipe_pop_ = μ_wipe_/σ_wipe_). We used three different CVs–1.0, 2.0, and 4.0–in order to generate synthetic wipe concentrations for a subject's home *i* on a day 1 (C_wipe,i,1_). We estimated parameters (*a*, mean and *b*, standard deviation) of the associated normal distribution, ln (C_wipe,i,1_), by the following method of moments [24].
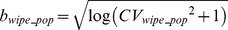
(1)

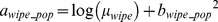
(2)where *a*_wipe_pop_ and *b*_wipe_pop_ are the mean and standard deviation of ln (C_wipe,i,1_), respectively. We then used the following lognormal inverse cumulative distribution function (cdf) to generate wipe concentrations for 500 homes with a residential receptor population for the first day of exposure.
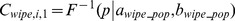
(3)where *C*_wipe,i,1_ is the wipe concentration selected with probability of *p* from the inverse lognormal cdf with parameters *a*_wipe_pop_ and *b*_wipe_pop_ for a subject's home *i* on a day 1. Since the wipe concentration for a subject's home *i* on a given day *j* (C_wipe,i,j_) is likely to be correlated to that on a previous day (C_wipe,i,j-1_), we used a log-Gaussian random walk to generate auto-correlated C_wipe,i,j_. In other words, we first generated random numbers that are log-normally distributed using mean (μ = 0) and standard deviation (σ_wipe_day_ = 1.0, 2.0, and 4.0). Then, we randomly multiplied 1 or −1 by the randomly generated numbers and computed cumulative sums. This allows us to approximate the temporal autocorrelation expected for the same house from day to day. In addition, since wipe concentrations should be positive, they were scaled up to assure positive values, maintaining the distribution of concentrations from random walk.

The method to generate ‘home’ and ‘food’ exposures is the same, but the simulated numbers are different as we used a random number generator for each exposure source. Thus, for food exposures, Equations 1 through 3 are used to generate food exposures for each simulated subject *i* on a given day *j* (E_food,i,j_) by replacing μ_wipe_, σ_wipe_day_, and CV_wipe_pop_ with μ_food_, σ_food_day_, and CV_food_pop_. Auto-correlated wipe concentrations and food exposures are provided in Figure S1.

#### 2. 2. 2. Biological concentrations

Because we assumed that the biological levels result from different combinations of average home exposure (E_home_) and food exposure (E_food_), we computed the relative E_food_ to E_home_ ratio using the following equation.
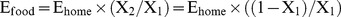
(4)where *X*_1_ and *X*_2_ are the percent contribution of exposure from home and food, respectively. Here, because we assumed that total exposure (E_total_) is equal to the sum of E_home_ and E_food_, the sum of *X*_1_ and *X*_2_ is 100%.

Using the different contributions to exposure from the home (X_1_), we added E_home,i,j_ and E_food,i,j_ to obtain a total daily intake rate for a subject *i* on a given day *j* (I_i,j_). Then, we used the one-compartment pharmacokinetic model described in Equation 5 to estimate time-dependent biological concentrations [3], [25] using serum as the representative biological medium. 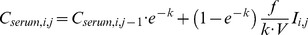
(5)where *C*_serum,i,j_ is the serum concentration of the compound for a subject *i* at time *j* (µg/L), *k* is an excretion rate coefficient of the compound (1/day), *f* is the fraction of the ingested compound present in the blood after absorption across the gastrointestinal tract and distribution throughout the body (unitless), *V* is the volume of blood (L), and *I*_i,j_ is the intake rate of the compound for a subject *i* at time *j* (µg/day), summed from E_home,i,j_ and E_food,i,j_.

In this model, the excretion rate coefficient *k* can be expressed as ln(2)/t_1/2_ where t_1/2_ is the half-life of the compound in the human body. We assumed that the fraction *f* is assumed to be 1 for all compounds and the blood volume *V* is about 5 L for all subjects [26]. This approach can be applied for urine concentrations and can be adjusted as needed.

### 2. 3. Sensitivity Analysis

Identifying the most important sources of overall exposure variability allows researchers to concentrate resources on obtaining the most important exposure data [6]. Thus, we conducted a sensitivity analysis to determine which sources of variability have relatively more influence on the R^2^ value for a given home-exposure contribution. Four types of exposure variability, σ_wipe_day_, CV_wipe_pop_, σ_food_day_, and CV_food_pop_, were considered in our study. We computed the mean R^2^ for compounds with short and long half-lives by varying one exposure variability (e.g., CV_wipe_day_) from 0.2 to 4.0, but fixing other exposure variability at 1.0 and then repeated this computation for other variability.

## Results

### 3. 1. Correlation Coefficient and Home Exposure Contribution

**Figure 1:**
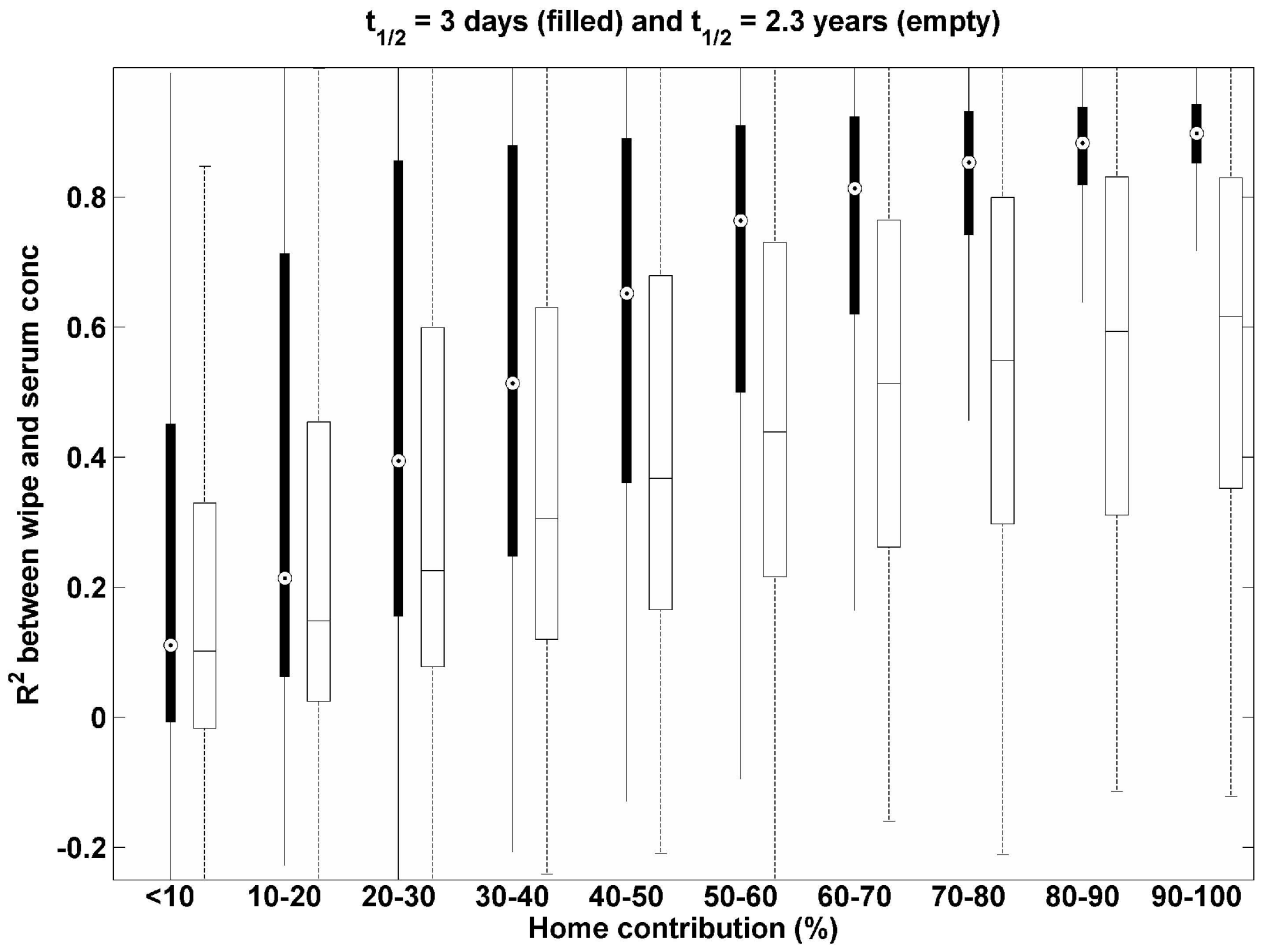
R^2^ between wipe and serum concentrations with different contribution of home exposure for two compounds with 3 days of half-life (filled) and 2.3 years of half-life (empty).

In this study, we applied various exposure scenarios to investigate the relationship between R^2^ and a relative contribution of home exposure to total exposure for compounds with different biological half-lives. Figure 1 shows that the R^2^ between wipe and serum concentrations increases with the increasing contribution of home exposure. Overall, as the home contribution increases, the gap between the median R^2^ for a long half-life compound (empty box) and that for a short half-life compound (filled box) increases. In addition, the median R^2^ is almost always larger for a compound with a short biological half-life compared to a compound with a long half-life when these compounds have the same average exposure contribution from the home environment. This is because biologic concentrations for the compound with a short half-life are more sensitive to home exposure with large variance, while concentrations for the compound with a long half-life remain relatively stable due to the longer body retention of the compound, which to a large extent buffers the variations. This result also indicates that for compounds primarily associated with food-based exposure, in other words, for those with little contribution from home exposure (e.g., BPA and outdoor use pesticides) [1], [18], [20]–[23], the R^2^ value becomes very small, as expected. In addition, for compounds with a large fraction of exposure resulting from indoor residential environments, such as PBDEs, the median R^2^ at 90–100% of home contribution is approximately 0.6 [4], [14]–[17] as shown in Figure 1.

**Figure 2:**
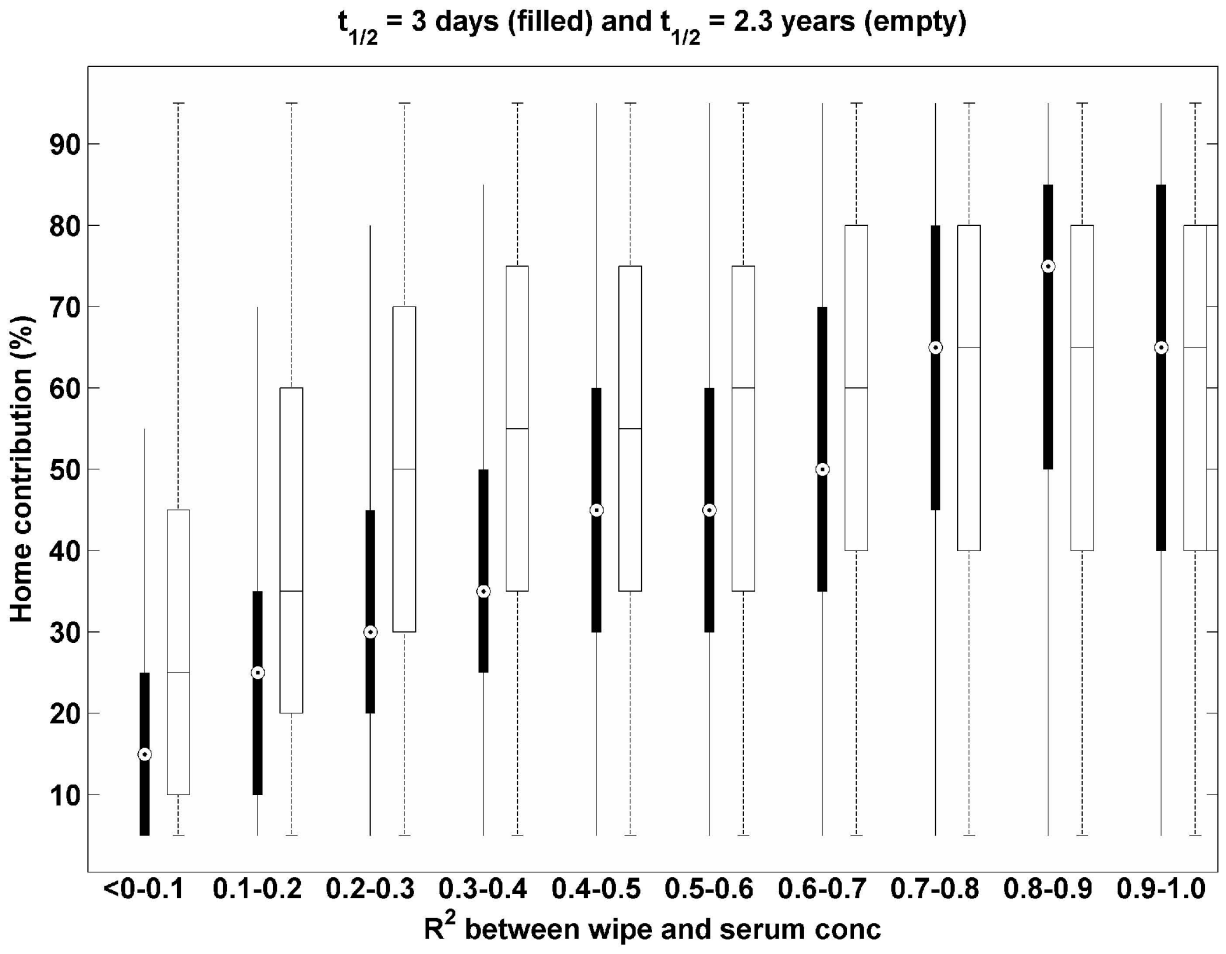
Contribution of home exposure (%) to total exposure with different R^2^ for two compounds with 3 days of half-life (filled) and 2.3 years of half-life (empty).

To look at the results in Figure 1 in a different point of view, we plot the percent of home exposure contribution with different R^2^ values to reveal the relationship between the biological half-life of the compound and the relative contribution of home exposure in Figure 2. This figure illustrates that, although the R^2^ between wipe and serum concentrations for two compounds with different half-lives is within the same range, the relative contribution of home exposure to total exposure differs by up to 20% between compounds. For example, when the R^2^ values for two compounds with different half-life values is between 0.3 and 0.4, the resulting contribution from the home environment for the short half-life compound is 20% smaller than that for the long half-life compound.

**Figure 3:**
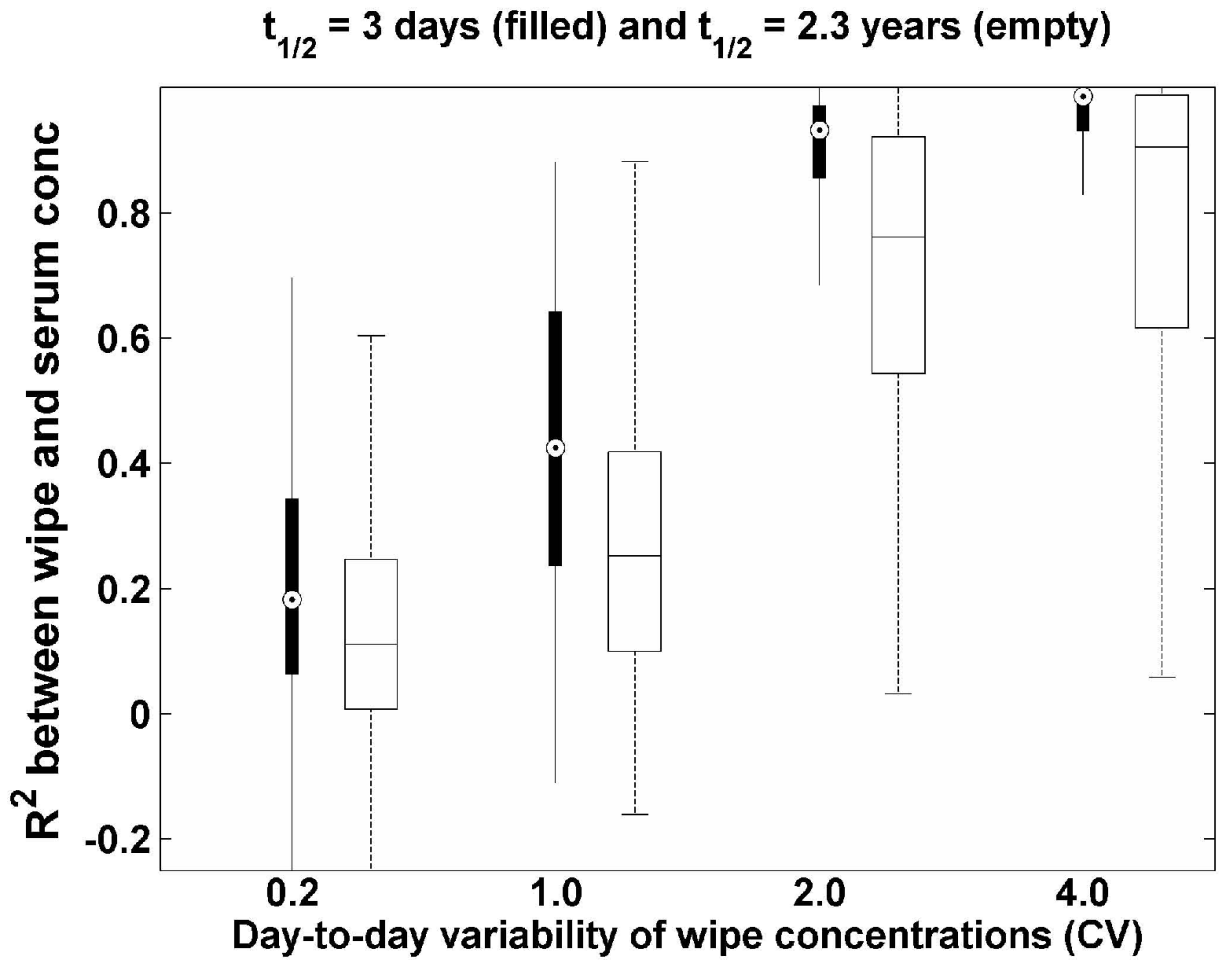
R^2^ with different day-to-day variability of wipe concentrations for two compounds with 3 days of half-life (filled) and 2.3 years of half-life (empty).

In actual exposure situations, we expect the day-to-day variability of wipe concentrations for semivolatile organic compounds to be small, due to their strong persistence on surface materials and dust [27]. In this study, we did not include the day-to-day variability associated with the relationship between the concentration in the home environment and the resulting exposure. There are two basic models for relating home concentrations to exposure. First, there are models that assume that exposure is driven by direct surface contacts, which are likely to have high day-to-day variability [28]. Second, there are models that assume that air-to-skin trans-dermal uptake becomes more significant than dermal uptake from surface contacts, and air-to-skin transfer is likely to be less variable day to day [12], [29]. These model choices are important because R^2^ values will also be linked to whether intake is primarily associated with air-to-skin trans-dermal uptake or dermal uptake from surface contacts. Thus, under the same conditions used in Figures 1 and 2, but with equal contributions from home and food (X_1_ = X_2_ = 0.5), we also investigated relative changes of R^2^ with different day-to-day variability of wipe concentrations (i.e., σ_wipe_day_) with results shown in Figure 3. The gap between the median R^2^ for a long half-life compound (empty box) and a short half-life compound (filled box) increases with increasing σ_wipe_day_. For all values of σ_wipe_day_, the median R^2^ for a compound with a short half-life is larger than that with a long half-life. This result indicates that day-to-day variability of wipe concentrations determines not only the magnitude of R^2^ for both compounds, but also the relative magnitude of R^2^ between compounds.

### 3. 2. Influential Source of Variability on Correlation Coefficients

We determined the sensitivity of the mean R^2^ value to each of the four different types of variability (i.e., σ_wipe_day_, CV_wipe_pop_, σ_food_day_, and CV_food_pop_) across a range of scales of variability (e.g., CV = 0.2, 1.0, 2.0, and 4.0). Table 1 shows the mean R^2^ at a specific variability for two compounds with different biological half-lives (t_1/2_). In terms of changes in mean R^2^ between compounds, a compound with a 2.3 year half-life is shown to be less sensitive to day-to-day variability of food concentrations (i.e., σ_food_day_) than one with a 3 day half-life and both compounds have similar sensitivity to day-to-day variability of wipe concentrations (i.e., σ_wipe_day_). In terms of changes in mean R^2^ within compounds, R^2^ is most sensitive to day-to-day variability of wipe concentrations for both compounds. In addition, the contributions of population variability of wipe concentrations and food exposures to changes in mean R^2^ are minimal.

**Table 1:** Mean R^2^ at a specific variability for four types of variability (coefficient of variation (CV) or standard deviation (σ)) for two compounds with different half-lives (t_1/2_).

	t_1/2_ = 3 days					t_1/2_ = 2.3 years				
σ or CV	0.2	1.0	2.0	4.0	range	0.2	1.0	2.0	4.0	range
σ_wipe_day_	0.36	0.71	0.91	0.94	0.36–0.94	0.20	0.44	0.69	0.80	0.20–0.80
CV_wipe_pop_	0.72	0.71	0.72	0.75	0.71–0.75	0.45	0.44	0.45	0.50	0.44–0.50
σ_food_day_	0.84	0.71	0.44	0.13	0.13–0.84	0.52	0.44	0.30	0.07	0.07–0.52
CV_food_pop_	0.71	0.71	0.73	0.68	0.68–0.73	0.43	0.44	0.47	0.42	0.42–0.47

### 3. 3. Implications/Limitations

Because some indoor contaminants are considered potential threats to human health, many studies have applied significant resources to examine the relationship between exposure to indoor pollutants and adverse health effects. However, these studies are potentially limited by the use of a single or a few environmental and biological samples. The significant implications of this situation are reflected in our results. Multi-day, multi-person sample analyses are costly and labor-intensive. In addition, the resulting R^2^ values from these studies are not interpreted or poorly interpreted in terms of variability and contribution of exposure sources and the biological half-life of a compound. In this regard, the simulation study in this paper provides an important step towards interpreting the relative contribution of home-based exposure to human body burden for two compounds whose biological half-lives are significantly different (days versus years). Although these two compounds do not cover the full range of chemical substances, bracketing half lives allows us to quantify the significance of source, measurement, and exposure pattern variability for disaggregating body burden. In particular, it shows that exposure variability and different contributions of exposure sources are more interconnected than commonly considered in many experimental studies. The work also brings to attention the need to understand the impact of a chemical half-life on the relationship between environmental exposures and biomonitoring data. The sensitivity of day-to-day variability of wipe concentrations and food exposures on the resulting R^2^ values also points to the importance of understanding variability and contribution of exposure sources. Finally, future work includes computing the relative number of samples needed for various levels of confidence to disaggregate body burden for various types of compounds (half lives), environments, and exposure pathways.

Despite the lack of experimental data, the simulated results provide key insights on the role of the variability and contribution of exposure sources and biological half-lives in quantifying a relationship between indoor exposure and human body burden. This approach will be useful for designing future exposure and epidemiologic studies that includes indoor environmental samples and biomonitoring data.

## Supporting Information

Figure S1 **Randomly selected example of auto-correlated wipe concentrations (top) and food exposures (bottom) from log-Gaussian random walk.**
(DOC)

## References

[1] Bradman A, Whitaker D, Quiros L, Castorina R, Henn BC, et al (2007) Pesticides and their metabolites in the homes and urine of farmworker children living in the Salinas Valley, CA. J Expo Sci Env Epid 17: 331–349. 10.1038/sj.jes.7500507 16736054

[2] Fraser AJ, Webster TF, Watkins DJ, Nelson JW, Stapleton HM, et al (2012) Polyfluorinated Compounds in Serum Linked to Indoor Air in Office Environments. Environ Sci Technol 46: 1209–1215. 22148395 10.1021/es2038257 PMC3262923

[3] Shin HM, Vieira VM, Ryan PB, Steenland K, Bartell SM (2011) Retrospective Exposure Estimation and Predicted versus Observed Serum Perfluorooctanoic Acid Concentrations for Participants in the C8 Health Project. Environ Health Perspect 119: 1760–1765. 21813367 10.1289/ehp.1103729 PMC3261988

[4] Watkins DJ, McClean MD, Fraser AJ, Weinberg J, Stapleton HM, et al (2011) Exposure to PBDEs in the Office Environment: Evaluating the Relationships Between Dust, Handwipes, and Serum. Environ Health Perspect 119: 1247–1252. 21715243 10.1289/ehp.1003271 PMC3230398

[5] Chen CC, Wu KY, Chang MJW (2004) A statistical assessment on the stochastic relationship between biomarker concentrations and environmental exposures. Stoch Env Res Risk A 18: 377–385.

[6] Loomis D, Kromhout H (2004) Exposure variability: Concepts and applications in occupational epidemiology. Am J Ind Med 5: 113–122. 10.1002/ajim.10324 14691975

[7] Rappaport SM, Symanski E, Yager JW, Kupper LL (1995) The relationship between environmental monitoring and biological markers in exposure assessment. Environ Health Perspect 103: 49–53. 10.1289/ehp.95103s349 PMC1519016 7635112

[8] Centers for Disease Control and Prevention (CDC) (2005) Third National Report on Human Exposure to Environmental Chemicals. Atlanta, GA.

[9] Centers for Disease Control and Prevention (CDC) (2009) Fourth National Report on Human Exposure to Environmental Chemicals. Atlanta, GA.

[10] Shin HM, McKone TE, Bennett DH (2013) Evaluating environmental modeling and sampling data with biomarker data to identify sources and routes of exposure. Atmos Environ 69: 148–155.

[11] Griffith W, Curl CL, Fenske RA, Lu CA, Vigoren EM, et al (2011) Organophosphate pesticide metabolite levels in pre-school children in an agricultural community: Within- and between-child variability in a longitudinal study. Environ Res 111: 751–756. 21636082 10.1016/j.envres.2011.05.008 PMC3726011

[12] Shin HM, McKone TE, Bennett DH (2012) Intake Fraction for the Indoor Environment: A Tool for Prioritizing Indoor Chemical Sources. Environ Sci Technol 46: 10063–10072. 22920860 10.1021/es3018286

[13] Klepeis NE, Nelson WC, Ott WR, Robinson JP, Tsang AM, et al (2001) The National Human Activity Pattern Survey (NHAPS): a resource for assessing exposure to environmental pollutants. Journal of Exposure Analysis and Environmental Epidemiology 11: 231–252. 11477521 10.1038/sj.jea.7500165

[14] Johnson PI, Stapleton HM, Slodin A, Meeker JD (2010) Relationships between Polybrominated Diphenyl Ether Concentrations in House Dust and Serum. Environ Sci Technol 44: 5627–5632. 20521814 10.1021/es100697q PMC2917910

[15] Stapleton HM, Eagle S, Sjoedin A, Webster TF (2012) Serum PBDEs in a North Carolina Toddler Cohort: Associations with Handwipes, House Dust, and Socioeconomic Variables. Environ Health Perspect 120: 1049–1054. 22763040 10.1289/ehp.1104802 PMC3404669

[16] Watkins DJ, McClean MD, Fraser AJ, Weinberg J, Stapleton HM, et al (2012) Impact of Dust from Multiple Microenvironments and Diet on PentaBDE Body Burden. Environ Sci Technol 46: 1192–1200. 22142368 10.1021/es203314e PMC3268060

[17] Wu N, Herrmann T, Paepke O, Tickner J, Hale R, et al (2007) Human exposure to PBDEs: Associations of PBDE body burdens with food consumption and house dust concentrations. Environ Sci Technol 41: 1584–1589. 17396645 10.1021/es0620282

[18] Geens T, Aerts D, Berthot C, Bourguignon JP, Goeyens L, et al (2012) A review of dietary and non-dietary exposure to bisphenol-A. Food Chem Toxicol 50: 3725–3740. 22889897 10.1016/j.fct.2012.07.059

[19] Xu Z, Fiedler S, Pfister G, Henkelmann B, Mosch C, et al (2013) Human exposure to fluorotelomer alcohols, perfluorooctane sulfonate and perfluorooctanoate via house dust in Bavaria, Germany. Sci Total Environ 443: 485–490. 23220138 10.1016/j.scitotenv.2012.10.089

[20] Coronado GD, Vigoren EM, Thompson B, Griffith WC, Faustman EM (2006) Organophosphate pesticide exposure and work in pome fruit: Evidence for the take-home pesticide pathway. Environ Health Perspect 114: 999–1006. 16835050 10.1289/ehp.8620 PMC1513343

[21] Curwin BD, Hein MJ, Sanderson WT, Striley C, Heederik D, et al (2007) Urinary pesticide concentrations among children, mothers and fathers living in farm and non-farm households in Iowa. Ann Occup Hyg 51: 53–65. 16984946 10.1093/annhyg/mel062

[22] Quiros-Alcala L, Bradman A, Smith K, Weerasekera G, Odetokun M, et al (2012) Organophosphorous pesticide breakdown products in house dust and children's urine. J Expo Sci Env Epid 22: 559–568. 10.1038/jes.2012.46 PMC4133088 22781438

[23] Rothlein J, Rohlman D, Lasarev M, Phillips J, Muniz J, et al (2006) Organophosphate pesticide exposure and neurobehavioral performance in agricultural and nonagricultural Hispanic workers. Environ Health Perspect 114: 691–696. 16675422 10.1289/ehp.8182 PMC1459921

[24] Ramaswami A, Milford JB, Small MJ (2005) Integrated Environmental Modeling-Pollutant Transport, Fate, and Risk in the Environment. Hoboken, New Jersey: John Wiley & Sons. Inc.

[25] Bartell SM (2003) Statistical Methods for Non-Steady-State Exposure Estimation Using Biomarkers. Davis, CA: University of California, Davis.

[26] Lorber M, Egeghy PP (2011) Simple Intake and Pharmacokinetic Modeling to Characterize Exposure of Americans to Perfluoroctanoic Acid, PFOA. Environ Sci Technol 45: 8006–8014. 21517063 10.1021/es103718h

[27] Shin HM, McKone TE, Tulve NS, Clifton MS, Bennett DH (2013) Indoor Residence Times of Semivolatile Organic Compounds: Model Estimation and Field Evaluation. Environ Sci Technol 47: 859–867. 23244175 10.1021/es303316d

[28] Cohen Hubal EA, Sheldon LS, Burke JM, McCurdy TR, Berry MR, et al (2000) Children's exposure assessment: A review of factors influencing children's exposure, and the data available to characterize and assess that exposure. Environ Health Perspect 108: 475–486. 10.1289/ehp.108-1638158 PMC1638158 10856019

[29] Weschler CJ, Nazaroff WW (2012) SVOC exposure indoors: fresh look at dermal pathways. Indoor Air 22: 356–377. 22313149 10.1111/j.1600-0668.2012.00772.x

