# Supplementary material for: Tracking Contributions to Human Body Burden of Environmental Chemicals by Correlating Environmental Measurements with Biomarkers
Source: PLoS One. 2014 Mar 28;9(3):e93678. doi: 10.1371/journal.pone.0093678 (PMC3969314; doi:10.1371/journal.pone.0093678)
Supplement: Figure S1 — Randomly selected example of auto-correlated wipe concentrations (top) and food exposures (bottom) from log-Gaussian random walk. (DOC) [file pone.0093678.s001.doc]

**Figure S1**. Randomly selected example of auto-correlated wipe concentrations (top) and food exposures (bottom) from log-Gaussian random walk.
